# Comparison of the Rheological Behavior of Fortified Rye–Wheat Dough with Buckwheat, Beetroot and Flax Fiber Powders and Their Effect on the Final Product

**DOI:** 10.3390/foods12030559

**Published:** 2023-01-27

**Authors:** Greta Adamczyk, Zuzanna Posadzka, Teresa Witczak, Mariusz Witczak

**Affiliations:** 1 Department of Food Technology and Human Nutrition, Institute of Food Technology and Nutrition, University of Rzeszow, Zelwerowicza Street 4, 35-601 Rzeszow, Poland; 2 Department of Engineering and Machinery for Food Industry, Faculty of Food Technology, University of Agriculture in Krakow, Balicka Street 122, 30-149 Krakow, Poland

**Keywords:** rye–wheat dough, burger’s model, creep and recovery test, quality bread parameters

## Abstract

This study was focused on the replacement of the part of the flour (10% *w*/*w*) in rye–wheat bread with three different botanical origin powders with a high dietary fiber content (buckwheat hulls, beetroot and flax powder). The dough was based on rye–wheat flour without and with the addition of fiber powders with different botanical origins and was tested, and the quality of the finished baked products made from those doughs were assessed. In order to characterize the flour mixtures, their basic parameters were determined, and their pasting characteristic was performed. The dough parameters were described by the Burger rheological model and also the creep and recovery test. On the other hand, in bread, the basic parameters of baking, crumb and crust color parameters were determined, and an analysis of the crumb texture was carried out. Additionally, a sensory analysis of the finished products was carried out. The applied fiber additives influenced the pasting characteristics of the tested rye–wheat flour and were influenced by the dough rheological properties. It was found that used fiber powders changed the quality parameters of the final products. Despite this, using fiber at the amount of 10% as a flour substitute allowed us to obtain bread of a similar quality to the control sample.

## 1. Introduction

Bread is one of the most important components of the human diet. Cereal products are characterized by a high nutritional value, as they contain significant amounts of protein and provide macro- and micronutrients such as phosphorus, zinc, fluorine and B vitamins. The bakery production includes wheat, rye and mixed bread. Depending on the proportion of wheat and rye flour, mixed bread is divided into wheat–rye bread (contains more than 50% of wheat flour) and rye–wheat bread (has a larger share of rye flour) [1].

Rye (*Secale cereale* L.), after wheat, is the second most important cereal, used for the preparation of bread. The main bulk of rye bread production takes place in Russia, Poland, Germany, Finland, Ukraine and Denmark. Rye compared to wheat is much poorer in protein but richer in fiber content [2,3,4,5]. Moreover, the presence of fiber in bread is an important aspect of nutrition. Currently, there is a noticeable reduction in the consumption of bread and cereal products [6].

An important aspect regarding the quality and acceptability of bread is its freshness. Physicochemical changes causing a decrease in the quality of bread appear immediately after baking. This is related, among other things, to starch transformations, i.e., starch retrogradation, bread moisture changes or interactions of bread ingredients. These changes cause a decrease in the sensory quality and spatial structure of the crumb [7,8,9]. Consumers expect food products with a high nutritional value that provide additional health benefits through the incorporation of new ingredients, for example food by-products that are rich in fiber and bioactive compounds. This sort of natural product protects against certain diseases and helps maintain the physical intellectual ability, and this interest is clearly visible in the bread market. Consumers are aware of the role of bread in their diet more and more, and they often reach for food made from natural ingredients, and thus for organic bread, consequently resigning from the traditional products [10]. Bread is a good example of a food product in which the addition of food industry by-products can be used. In this context, bread is of great interest for research and the industry because of the development of new functional products [11]. The main component of wheat and rye flour is starch. This biopolymer plays the most important role in the physio-chemical and rheological properties of the dough [12]. In addition, the presence of other non-starch ingredients (protein, fat and fiber contents) significantly influences the dough’s thermal and rheological properties [13]. Knowledge of the rheological properties of flour and dough allows for an optimization of bread-making technology to improve the quality and taste of the final products [14].

Many researchers have enriched bread with fruits, vegetables, seeds and non-traditional types of flour. However, the by-products formed during grain processing (buckwheat hulls, oat bran, wheat bran and flax seeds waste) are also potential sources of functional compounds. These kinds of raw materials have unique chemical compositions and high biological values [15,16,17,18,19]. 

The by-products are also used to produce dietary fiber (DF) powders [20]. DF is obtained from milling by-products, and also fruit and vegetable wastes, and is mainly applied to bakery products [21,22]. Hulls of buckwheat seeds have been reported as a dietary fiber [23] and are also a rich source of natural antioxidants [24,25]. Also the beetroot processing residues are a source of DF and can be used as an additive in cereal products. Still in the literature there is limited information about beetroot fiber use in cereal baked products (cookies or bread). Mainly, beetroot residues are applied to cakes, biscuits, noodles or some extrudates [26,27,28,29]. The application of beetroot in fresh bakery products, such as bread, is rare. This topic was taken up by Kohajdová et al. (2018) [30]. They focused their investigation on the influence of the incorporation (2–10 mass%) of beetroot powder on the rheological properties of wheat dough, and the qualitative and sensory attributes of wheat rolls. The hulls of buckwheat seeds that are produced during the de-hulling process have shown an increased interest in renewable by-products, as they are promising raw materials that can be used in production as food additives [31]. Research on the use of buckwheat hulls in wheat bread was carried out by Hromadkova et al. (2007) [15]. In another research, the rheological properties and bread parameters are significantly affected by the level of replacement of wheat flour by additives such as oilseeds or additives with a high content of dietary fiber [16]. The optimal share of such additives in bread is about 10% in relation to the amount of flour in the recipe. This amount was used by Bajwa (1997) [32], where wheat flour was replaced by cottonseed. Rao and Vakil (1980) [33] used defatted peanut flour to change the water absorption capacity and extensibility of the dough with wheat flour. Similarly, the 10% replacement of wheat flour by defatted peanut flour altered the water absorption capacity and extensibility of the dough mix. Ahluwalia and Kuar (2001) [34] observed a decreased mixing time of the dough, which was caused by an increased fiber content.

Bearing in mind the fact that there is no information in the literature on the enrichment of rye–wheat bread with additives with a high content of fiber and their characteristics due to rheological properties, we conducted preliminary research on the share of these additives in the dough and the final product. Based on our previous preliminary studies and the literature [15,16,18,32,33,34], the variant with the addition of 10% of the three different fiber preparations in terms of the chemical compositions was selected. The aim of our research was to initially determine the effect of three types of fiber powders (buckwheat, beetroot and flax powder) on the rheological properties of dough and the quality parameters of rye–wheat bread. Our research has not only a scientific aspect, but also an application aspect for regular industrial production, and at the same time, there is the possibility of obtaining a product that could be accepted by consumers.

## 2. Materials and Methods

### 2.1. Materials

Material for bread preparation consisted of commercial white rye flour type 720 (Melvit, Poland) (fat 1,7 g/100 g; carbohydrates 71 g/100 g; fiber 6 g/100 g; protein 6 g/100 g, ash 0.62 g/100 g), commercial white wheat flour type 650 (Gdanskie Mlyny, Poland) (fat 1,6 g/100 g; carbohydrates 68,8 g/100 g; fiber 3,5 g/100 g; protein 11 g/100 g, ash 1 g/100 g), buckwheat fiber (BH) (LookFood, Poland) (total dietary fiber (TDF) 71.40 g/100 g, fat 0.60 g/100 g; carbohydrates 3.10 g/100 g, protein 4.50 g/100 g); beetroot fiber (BR) (LookFood, Poland) (fat 0.3 g/100 g; carbohydrates 64.0 g/100 g, fiber 16.1 g/100 g, protein 9.8 g/100 g), and flax fiber (FL) (LookFood, Poland) (fat 13.4 g/100 g; carbohydrates 4.8 g/100 g; fiber 35.7 g/100 g, protein 33.8 g/100 g).

### 2.2. Methods

#### 2.2.1. Basic Parameters of Flour and Dough

In the used flour blends (rye–wheat and rye–wheat with BH, BR and FL) the moisture content was determined (%) using a MAC-50 moisture analyzer (Radwag, Poland), and the water absorption and dough properties (dough development time, dough stability time, dough softening in Farinograph Units [FU] and farinograph quality number) were developed using a Farinograph-E (Brabender GmbH & Co. KG, Duisburg, Germany), in accordance with ICC Standard No. 115/1 [35]. The test was carried out on the mass of flour blend equivalent to 300 g of flour with a moisture content of 12%. The falling number was measured using a Falling Number 1900 System (Perten Instruments, Hägersten, Sweden) by ICC Standard No. 107/1 [36]. All tests were performed in triplicates. Additionally, flour blends were examined for protein and ash content using a DA 7200 NIR analyzer (Perten Instruments, Germany) equipped with a stationary monochromator and a diode array detector. 

#### 2.2.2. Pasting Characteristics of Flour Blends

The impact of fiber addition on gelatinization characteristics of the studied formulations of flour blends was checked by Viscograph-E Brabender (Brabender GmbH & Co. KG, Duisburg, Germany), running at 75 rpm and operating under Viscograph Data Correlation software, Brabender GmbH & Co. KG (Duisburg, Germany). Dry blends (80 g studied formulations) without yeast suspended in 450 mL of water were heated/cooled at a rate 1.5 °C/min according to the following program: rising temperature in the range 30–95 °C, constant temperature 95 °C (5 min) and cooling in the range 95–30 °C. Measurements were run in duplicates. Based on the analysis, the following parameters were obtained: T_0_ (°C)—the temperature at the beginning of pasting; η_max_ (Brabender units (BU))—maximum viscosity; T_ηmax_ (°C)—temperature at maximum viscosity; η_95°C_ (BU)—viscosity at 95 °C; BD (BU)—breakdown; SB (BU)—setback; η_30°C_ (BU)—viscosity after cooling to 30 °C. Viscograph Data Correlation software was used to process the data (Brabender GmbH & Co. KG, Duisburg, Germany). 

#### 2.2.3. Preparation of Rye–Wheat Dough and Baking Process

The dough was prepared from flour mixture consisting of commercial white rye flour (type 720) and commercial white wheat flour (type 650), where in tested samples, 10% (in relations to the flour mixture by weight) was replaced with commercial dietary fibers: buckwheat, beetroot and flax fiber powders (BH, BR and FL). The control sample was prepared from only rye–wheat flour mixture (Table 1). The bread was made with flour mixture, with or without dietary fiber preparation, baker’s yeast (3% by weight of flour mixture used) and water, using laboratory mixer (Mesko-AGD, Poland). After mixing, the doughs were fermented for 60 min at 30 °C, with a simultaneous puncture after 30 min. Afterwards, dough pieces of 250 g weight were formed and placed into molds. The final dough fermentation lasted 30 min. The breads were baked using an electric modular oven (Sveba Dahlen, Fristad, Sweden) at 230 °C for 30 min [37,38].

#### 2.2.4. Rheological Properties of the Dough Samples

The rheological characteristics of dough samples were performed at a temperature of 25 °C using a rheometer MARS II (Thermo-Haake, Germany) in oscillatory mode with parallel-plate geometry (diameter 35 mm) and 1 mm gap. The dough samples were placed in the measuring system and left for 5 min to allow for the relaxation and stabilization of the temperature of the samples.

##### Oscillatory Measurements

The range of linear viscoelasticity was determined by examining, at a constant angular velocity of 1 rad/s, the dependence of the modulus: storage (G′) and loss (G″) on the stress in the range 0.1–1000 Pa. The mechanical spectra (G′ and G″), within the range of the linear viscoelasticity region for all samples, were determined at constant strain amplitude (γ = 0.0003) in the angular frequency range of 0.1–100 rad/s [39].

The obtained experimental data were described by the power-law equations:

G′(ω) = K′∙ω^n′^ (1)

G″(ω) = K″∙ω^n″^ (2)

where G′—storage modulus (Pa); G″—loss modulus (Pa); ω—angular frequency (rad/s); and K′, K″, n′, and n″—constants.

The mechanical spectra were run in triplicates.

##### Creep and Recovery Tests

The creep and recovery tests were performed in the range of the strain-to-stress ratio (determined based on linear viscoelasticity tests) at constant stress in the creep phase σ_0_ = 10 Pa. The creep step lasted 150 s and the recovery step 600 s. The experimental data were described using a four-parameter Burger mode [40,41].

The equation for the creep phase (3):

(3) $$J\left(t\right)=J_{0}+\frac{t}{\eta_{0}}+J_{1}\cdot (1-\exp^{-\frac{t}{\lambda_{ret}}})\;\;\;\;$$

The equation for recovery phase (4):

(4) $$J\left(t\right)=\frac{t_{1}}{\eta_{0}}-J_{1}\cdot (1-\exp^{\frac{t_{1}}{\lambda_{ret}}})\cdot \exp^{-\frac{t}{\lambda_{ret}}}\;$$

where J(t) is a compliance (Pa^−1^); J_0_—instantaneous compliance; J_1_—retardation compliance (Pa^−1^); η_0_—zero-shear viscosity (Pa∙s); λ_ret_—retardation time (s) and t_1_—time after which stress was removed (s). 

The calculations were performed by Marquardt–Levenberg’s method, using the non-linear estimation module of the Statistica 12.0 program (StatSoft Inc., Tulsa, OK, USA).

#### 2.2.5. Laboratory Baking Parameters

Twenty-four hours after baking, the parameters for the laboratory-produced breads were determined, such as dough yield (%), bread yield (%) and baking loss (%) [42,43]. Moreover, the volume of bread (cm^3^) was determined using the Sa-Way apparatus, using loose millet seeds [43,44]. The specific volume, i.e., the volume of bread per 1 g of flour used (cm^3^/g) [45], was also calculated.

#### 2.2.6. Bread Color

The color of the bread crumb and crust was determined using spectrophotometer (HunterLab, Reston, VA, USA). The results were expressed according to the CIEL*a*b* color system, with reference to illuminant D65 and a visual angle of 10°. The following parameters were determined: lightness (L*), redness+/greenness (a*), and yellowness+/blueness (b*). Arithmetic means from three repetitions were calculated for each type of wheat bread [46].

#### 2.2.7. Texture Profile

The analyses of the crumb texture (TPA) of the tested breads were performed using the testing machine EZ Test EZ-LX supported by Trapezium X Texture PI software (Shimadzu, Kyoto, Japan). The bread samples were compressed twice to 50% deformation using a probe with a diameter of 25 mm and a speed 50 mm min^−1^. The period between the cycles was 5 s. The following parameters were determined: hardness (N), cohesiveness (-), chewiness (N), gumminess (N) and resilience (-). The determination was performed in four replications [47].

#### 2.2.8. Sensory Breads Quality

Sensory breads quality was evaluated in accordance with the Polish standard for wheat bread (PN-A 74108:1996), described by Diowksz and Sadowska (2021) [48]. The sensory analysis involved the following parameters: appearance of a loaf, crust and crumb, and also crumb flavor. Each parameter was awarded with total scores as either “very good” I (32–28), “good” II (27–23), “satisfactory” III (22–18) or “poor” IV (17–0). Breads were qualified by a sensory panel aged 28–55 (5 women and 5 men) into a proper quality level (I, II, III and IV) according to obtained scores.

#### 2.2.9. Statistical Analysis

The experimental data were calculated using Statistica v.13.3 (TIBCO Statistica Inc, Palo Alto, CA, USA). The analysis of variance was performed using Duncan’s test at the confidence level of α = 0.05. Additionally, the values of Pearson’s correlation coefficients between selected parameters characterizing dough properties and bread quality parameters were calculated.

## 3. Results and Discussion

### 3.1. Basic Parameters of Flour and Dough

The results of the determination of the flour moisture, protein content, ash content, falling number and the water absorption of the flours, and the farinographic analysis of the doughs are presented in Table 2.

The tested samples of the flour mixtures were characterized by the moisture content in the range of 12.31–12.68%. The highest value of moisture was for the control sample (rye–wheat blend). The control sample composite from rye and wheat flour had 10.6% of protein, and replacing this flour with buckwheat fiber significant reduced the amount to 9.3%. Other fibers, beetroot and flax, caused an increase in the protein content in designed flour blends to 11.8% and 13.3%, respectively. The ash content in the rye–wheat flour mixture (0.89%) increased with the share of fiber additives used in the recipes, and the highest was observed in the case of the addition of buckwheat fiber, where the increase in content was almost threefold.

The falling number test is a method of assessing α-amylase activity in flour, and the higher the falling number, the lower the amylase activity. A falling number is an indicator of the wheat flour quality, but its use also as an indicator of the rye flour quality is still being explored. The falling number below 250 in wheat has a negative impact on the bread quality. The best range for wheat is 250–350. Higher falling numbers than 400 s can influence slower fermentation, a drier bread texture or a poorer loaf volume in the final product. Rye bread ferments more quickly than wheat, so in this case, it is expected that lower falling numbers are preferable for rye than for wheat (150–200 s). Low falling numbers can be caused by extended-harvest rye grains [Darby, 2022]. The falling number of the tested baking blends was significantly higher (227–244 s) compared to the flour without the addition of a high-fiber raw material (163 s) (Table 2). However, it should be noted that the tested bread had rye–wheat flour in the recipe. Thus, the obtained falling number values in the bread with the addition of fiber powders indicates good-quality products, because these values are close to 250 s.

The chemical composition determines the properties of the rye flour and dough. Flour composition differences are likely related to the operation of the roller mills and the increase in bran contamination. The water absorption and development time increased as the milling proceeded to the detriment of the peak time [49]. According to the research of other authors [50], the diverse botanical origin of the high-fiber additives to the wheat dough significantly affects the farinographic properties of the dough. Dough that was enriched with cellulose-rich fiber was generally characterized by a higher water absorption and dough stability [51]. The water absorption of the tested rye–wheat blend was 58.5%. Voicu et al. (2012) [52] showed that increasing the water absorption of the flour mixture was noted with an increase in the proportion of rye flour, and the greater value of this parameter, in the case of a blend with 30% share of rye flour, was 67%. In our research (Table 2), a diversified effect of the fiber preparations on the water absorption of the blends tested was noted, because supplementation with buckwheat and beetroot fibers decreased the water absorption of the flour blends. Conversely, a significant increase in water absorption was noted in the tests for the blend of flour with a flax fiber addition. This relationship is known from previous studies [53,54], in which there was a significant increase in the water absorption of wheat flour by several percent with the participation of flax seed products. The water absorption of the dough depends not only on the type of fibers, including their botanical origin, but is also related to characteristics such as the water-binding capacity and the interaction with gluten and other ingredients of the dough, which are very important [55]. In our discussed studies, the development time of the dough made from the control blend and the blend with the addition of flax fiber was undifferentiated, while the share of buckwheat fiber contributed to a shorter development time, and the share of beetroot fiber contributed to an extension of the dough development time. The consistency of the dough made from a rye–wheat blend with buckwheat and beetroot fiber was similar or higher compared to the control sample (rye–wheat without addition). This relationship was noted in the studies of other authors [56]. At the same time, the share of flax fiber in the tested blend significantly reduced the stability time of the dough obtained. The effect of flaxseed products on the dough made with their participation was noted in the study by Xu et al. (2014) [53]. The comparison of the farinograph quality number (Table 2) showed that the share of the fiber addition of buckwheat and beetroot improved the quality of the rye–wheat dough. Similar results from the farinographic analysis of wheat dough with added fiber were also obtained by Liu et al. (2019) [57], who concluded that, during mixing, the fiber-fortified doughs had difficulty reaching optimal states quickly, as evidenced by the longer development time and shorter stability time. At the same time, these studies also reported a surprisingly higher degree of dough weakening, while our results confirm a positive effect of fiber addition on dough softening (Table 2). The water absorption of dough depends on the nature of fibers not only in their capacity to bind to water, the interactions with gluten and other dough components, which are very important. This interaction changes all the farinographic characteristics of doughs.

### 3.2. Pasting Characteristics of Dough Mixes

Replacing part of the rye–wheat flour with BH, BR and FL modified the pasting properties of the sample suspensions. The pasting temperatures (T_0_) of the dough samples were in the range 55.4–58.1 °C (Table 3). The control sample started the gelatinization process at 56.0 °C, and an addition of buckwheat and beetroot powders caused an increase of this value to 58.1 and 57.1 °C, respectively. The opposite effect was observed with flax powder, where the value decreased significantly (55.4 °C). Czubaszek et al. (2021) [58] observed that a 10% replacement of rye flour with a brewer’s spent grain (barley and barley with buckwheat) rich in fiber caused a significant increase in the initial pasting temperature. The presence of BH and BR fiber powders in flour suspensions decreased the maximum viscosity (η_max_) from 295.0 BU (control sample) to 234.0 BU (BH) and 234.0 BU (BR), while FL powder increased this value to 656.0 BU. The high value of the maximum viscosity (656.0 B.U.) in the sample containing flax fiber is due to the high fat and protein content in the added fiber preparation and the formation of mucilage derived from the flaxseed [59]. The maximum viscosities of the tested samples were reached in the temperature range of 71.8–74.5 °C (T_η max_). At the maximum temperature (95 °C) during the measurements, there was a significant decrease in the viscosity (η_95°C_) of the tested samples. The greatest decrease in viscosity was noted in the control sample (9-fold), while in the remaining samples, it was about five–six-fold decrease. The breakdown parameter (BD) predicts the tendency of starch to resist shear force during the heating and stability of the final product. In the tested mixtures of rye–wheat flour (BD = 296.0 BU), the presence of BH and BR powders decreased this parameter to 192.5 and 204.0 BU, whereas FL powder increased the BD to 550.5 BU. The setback parameter (SB) had the lowest value in the control sample, but the highest in the flour with the flax powder addition. The flour with FL was characterized by a high final viscosity (η_30°C_) as well. 

The presence of protein as well as fat affected the pasting characteristics of the rye–wheat flour. Among the tested samples, the highest content of protein and fat was introduced with the flax powder (33.8 and 13.4 g/100 g of powder, respectively). The BH and BR samples contained less than 1% of fat and 4.5 g and 9.8 g of protein, respectively, in 100 g of the samples. In the sample with flax powder at the maximum viscosity, the breakdown and setback parameters were the highest among the tested samples. This means that the final product can be more stable. Moreover, the final viscosity (η_30°C_) of the mixture was high (503.5 BU). According to Ktenioudaki et al. (2010) [60] and Zaidul et al. (2004) [61], the pasting properties and rheological properties of wheat flour depend on the protein content in the sample. They indicated a correlation between the baking quality, dough strength, volume and textural attributes. 

### 3.3. Rheological Measurements of Doughs

#### 3.3.1. Oscillatory Measurements

**Figure 1:**
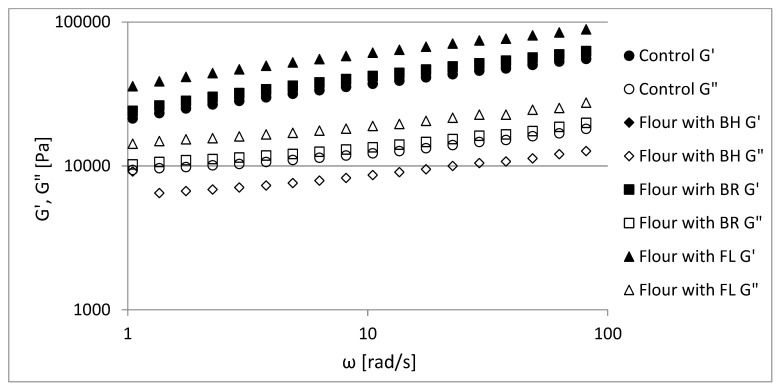
Mechanical spectra of dough samples for flour (control), flour with buckwheat (BH), beetroot (BR) and flax (FL).

The dough represents a viscoelastic material with non-linear properties [62]. Figure 1 shows the mechanical spectra of rye–wheat dough with and without fiber additives samples, which indicates that the storage (G′) and loss (G″) moduli increased with an angular frequency (ω). The mechanical spectra were obtained in the range of linear viscoelasticity. The ratio of the loss modulus to the storage modulus (G″/G′) provides insight into the elastic and viscous character of the food samples. When the storage modulus is greater than the loss modulus, the food material is interpreted with predominantly elastic properties [36,63,64]. The samples had G′ > G″ and tan δ from 0.40–0.44 (Table 4) (tan δ <1), implying the prevalence of elastic features over viscous. These results (G′ depends on ω and tan δ >0.1) showed properties typical for weak gels and were similar to the results obtained by Korus et al. (2009) [65] and Lazaridou et al. (2007) [40] of gluten-free dough formulations with hydrocolloids. The partial replacement of rye–wheat flour with buckwheat husk powder, beetroot and flax powder resulted in slight change of tan δ, from 0.44 to 0.41, 0.42 and 0.40, respectively.

The power-law equation parameters were tabulated in Table 4. The lowest values of the coefficients K′ and K″ were in the control sample (22,399 Pas^n′^ and 8774 Pas^n′^) and were the highest for the dough with flax powder (FL) (37,471 Pas^n′^ and 13,590 Pas^n′^). The differences between the control sample and formulations with BH were statistically insignificant (22,399 Pas^n′^, 23,395 Pas^n′^ and 8774 Pas^n′^, 8672 Pas^n′^). The values of n′ and n″ were statistically insignificant in all the studied samples. Some authors, who studied wheat dough and other doughs, such as a gluten-free dough with an addition of resistant starch and some hydrocolloids, also obtained n′ close to 0.20–0.25 [65,66,67].

#### 3.3.2. Creep and Recovery Test

**Figure 2:**
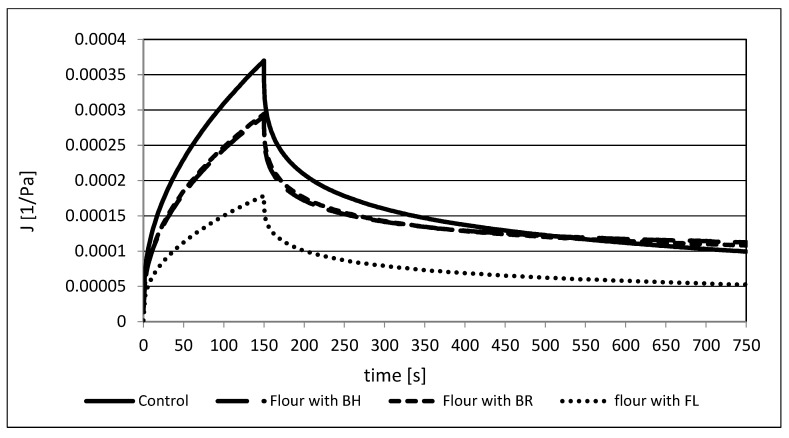
Creep and recovery curves of tested doughs.

The creep and recovery test is also useful to observe the viscoelastic properties of the dough. The typical creep curves are under constant stress for rye–wheat dough, and its mixtures with fiber-rich additives are shown in Figure 2. At zero stress part of stored energy is returned and can observe recovery phase. All curves have the shape characteristic of viscoelastic materials, and it is caused by reorientation of the bonds in this sort of material [68]. The addition of various powders rich in fiber significantly decreased the compliance values (J), and the greatest decrease was in the flax powder (FL) sample. In the case of BH and BR, changes were less evident, and the curves were almost the same.

Table 5 includes the parameters of the Burger’s model studied samples. The highest value of instantaneous compliance (J_0_) was observed in the control sample (0.107 × 10^−3^ Pa^−1^) and about a 50% lower value was observed in the dough with flax powder (0.053 × 10^−3^ Pa^−1^). In the case of dough with BH and BR, the values were lower by about 20% compared to the control. The replacement of rye–wheat flour with fiber powders also decreased the retardation compliance parameter (J_1_) from 0.248 × 10^−3^ Pa^−1^ (control) to 0.148–0.107 × 10^−3^ Pa^−1^ in the studied samples, where the lowest values was also in the sample with flax powder. The obtained trend of the decrease of these values was similar to those obtained by Lazaridou et al. (2007) [40] and Witczak et al. (2010) [69], whereby the authors modified gluten-free doughs using some hydrocolloids, resistant starch and maltodextrins in formulation. The retardation time (λ_ret_) of the studied samples was the highest in the control sample (129.4 s), but statistically insignificant changes of this parameter were found in the doughs with BH, BR and FL. The zero-shear viscosity (η_0_) had the greater value in the dough with FL (27.7 × 10^5^ Pa s) than in control sample (15.2 × 10^5^ Pa s). For samples with BH and BR, they were observed the lowest values of this parameter (12.8 and 13.4 × 10^5^ Pa s). Hüttner, Dal Bello and Arendt (2010) [70] studied the effect of different oat flours on the rheological properties of dough and the bread-making process. According to them, a major factor in the growth of elasticity is the water hydration capacity. Additionally, this factor depends on the sort of flour and protein content in the dough.

### 3.4. Laboratory Baking Parameters

The rye–wheat bread specific volume per 1 g had 2.04 cm^3^ (Table 6). The addition of fibers resulted in the decreased volume of the bread. The greatest volume loss was observed in the bread with the beetroot (1.87 cm^3^) and buckwheat additions (1.93 cm^3^). The same dependency was observed in the parameter loaf volume. Similar trends were indicated by Krochmal-Marchak et al. (2020) [47] in wheat bread, with various proportions of oat flour. Some authors [71] state that bread additives usually deteriorate the volume and texture parameters of bread. The replacement of rye–wheat flour with fiber additives causes reductions in the amount of gluten proteins in flour, which subsequently leads to a deterioration of the dough’s flexibility and bread texture. A decisive parameter of the bread baking value is its yield and total baking loss expressed in percent. The 10% addition of BR and BH fibers reduced the baking loss comparable to the control sample, while the FL did not. The lowest bread yield (139.43%) and the highest baking loss (9.51%) of bread was observed in the control sample. The baking losses, from a baking technology viewpoint, ought to be minimized as they are an unfavorable phenomena. They depend on the amount of water and volatility lost during baking. From a commercial point of view, the bread yield is an important parameter [72].

### 3.5. Color Parameters

The values of the color parameters (L*, a* and b*) of the crumb and crust of breads with additives rich in fiber are presented in Table 7. It was shown that breads enriched with these additives were characterized by a darker crumb compared to the control breads. The value of the L* parameter (defining the color component–brightness) of the bread with flax was about 15% lower than the control bread. The same relationship in the case of adding flax seeds to wheat–rye bread was observed by Kaszuba et al. (2017) [16], where this value also changed by about 15%.

In turn, the values of the L* parameter of the bread crumb with the addition of the beetroot preparation and buckwheat hulls decreased by about 40%. A change in the color of the crumb under the influence of the addition of seeds was found by Adamczyk et al. (2021) ([18]) and Coelho et al. (2015) [73], who observed a darkening of the crumb of wheat bread due to the addition of chia seed flour. Darkening of the bread crumb may be due to the increased amount of fiber and fat in the system due to the presence of the additives used, e.g., from oilseeds (flax), but also due to the dyes contained in these additives. The fiber preparation from the buckwheat husk had a brown color, while the beetroot introduced a red color to the bread. The parameter L* plays a significant role in bakery products. Due to the fact that the crumb does not reach as high a temperature during the baking process as the crust, the crumb color is similar to the color of the applied ingredients [74].

Therefore, in the obtained results, the value of parameter a* was the highest in the sample of bread with the addition of the beetroot powder (24.1), and significantly lower in the case of the addition of the flax powder (4.7) and buckwheat hulls (3.8). The value of parameter a of the control sample (3.5) did not have a statistically significant difference, only in relation to the sample with buckwheat hull (3.8). The addition of beetroot significantly increased a* values of crumb, where the red hues increased with the increasing levels of BR. According to Kohajdová et al. (2018) [30], this result may have been due to the original red–purple pigment of the beetroot. The obtained results showed that the parameter b* had the highest value in the case of the control sample (18.6), while the applied additives reduced its value to 17.2 (beetroot), 16.3 (flax) and 8.3 (buckwheat), respectively.

**Figure 3:**
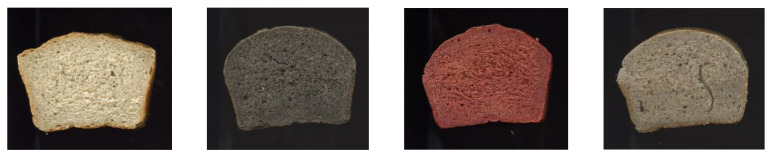
Rye–wheat breads, from left to right: control sample, bread with BH, BR and FL.

The crust color parameters of the tested breads differed from each other, as in the case of the crumb, which results from the different colors of the fiber additives (Figure 3, Table 7).

### 3.6. Texture Analysis

In the next research stage, the parameters of the texture of rye–wheat breads enriched with additives with a varied share of fibers were determined (Table 8). Hardness is the sample’s resistance to deformation until an external force is applied [75]. The hardness data showed that the share of the additives used in rye–wheat bread increased the hardness of the breadcrumbs, while the type of additives had no statistically significant effect on the value of the discussed parameter. Similarly, a statistical effect of the presence of buckwheat, beetroot and flax preparations on the consistency of the crumb was found. Consistency is the strength of the internal bonds, which make up the body of the product [75]. Bread without additives was characterized by the lowest gumminess and chewiness. The introduction of preparations with a high content of fiber into the recipe resulted in an increase in the value of these parameters in rye–wheat bread. Due to the tested level of the content of the fiber preparation, the proportion of the mixture fraction increases, additionally absorbing the water added during the preparation of the dough for baking, which results in it being more compact, less flexible compared to the control sample without the participation of the fiber preparation, and thus harder, i.e., less susceptible to any deformation.

According to Witczak et al. (2017) [76] and Hüttner et al. (2010) [70], a low dough viscosity positively affects the quality of bread, improving the expansion of gas cells during rising, ensuring a greater bread volume and structure with a well-aerated crumb. However, its excessive decrease may lead to a weakening of the structure, reduction of air and fermentation gas retention, and as a result, it may have a negative impact on the bread. In the analyzed case, statistically significant correlations were found only between the hardness and the degree of the softening of the dough (r = −0.985, *p* = 0.015), and between the dough yield and the zero-shear viscosity (η_0_) (r = 0.968, *p* = 0.032). It seems, therefore, that the relationship between the rheological parameters of the dough and the properties of the bread is not direct and depends on many factors.

### 3.7. Sensory Evaluation of Bread

The bread quality levels were based on the results of the sensory evaluation listed in Table 9. The highest total score of the sensory quality assessment was given for the control sample (27.0), where the appearance, crumb, crust and flavor had the best scores. On the other hand, the appearance of three products with fiber powders had the same level of the score (4.0). The products with fiber powders were regularly formed and arched. Such an effect in the appearance of bread with the addition of wheat fiber was obtained by Kučerová et al. (2013) [77]. Despite the intense color of bread with the addition of beetroot fiber (high value of the parameter a* = 24.1, Table 7, Figure 3) compared to the samples with flax and buckwheat fiber, the appearance was rated high and comparable to these samples. The obtained result indicates that, for the evaluation panel, the color could be associated with the use of an additive with health-promoting properties. In addition, the general appearance of the bread (shape, size) was comparable to the other studied samples, which is also confirmed by the results of the baking parameters (Table 6, Figure 3). Therefore, the evaluation panel gave the appearance a score of 4.0 for bread with buckwheat and flax fiber powders.

The crumb was the worst in the sample with the FL fiber (2.0) (was cracked), and the best in sample with BR (9.0) (Figure 3). The addition of the beetroot powder to rye–wheat bread caused a good-quality crust because the score was on the same level as the control sample (9.0). The products with an addition of BL and FL had good flavor (5.0) compared to the bread with BH. The flavor derived from buckwheat, i.e., bread with the addition of buckwheat fiber, received a low value of points (3.0).

Generally, the quality level II got bread without fiber, bread with BR fiber and quality-level III–bread with BH and FL.

## 4. Conclusions

In this research on rye–wheat bread, additives containing a high content of fiber were used. The research was carried out on the obtained raw doughs as well as on the final products. The use of 10% of fibers from buckwheat, beetroot and flax as a substitute for rye–wheat flour allowed us to obtain bread with good physical and chemical parameters. The rye–wheat dough, with the addition of a powder rich in fiber, behaved as a weak gel. The values of the storage modulus significantly depended on frequency. The values of the falling number in the samples of flour containing fiber powders (227–244 s) indicated the possibility of obtaining good-quality products [78]. This was confirmed by the received baking parameters of the final products, which slightly differed from those obtained in the control sample. It was found that the fiber powders used changed the quality parameters of the final products. As a result of increasing the share of fractions in mixtures that absorb water (the fiber fraction), the obtained products were characterized by a more compact crumb and lower flexibility compared to the control sample, and were thus, harder, and more brittle. However, despite the use of beetroot fiber, which gave the bread an intense color (a high value of the a* parameter), it did not disqualify this product during the sensory evaluation. This bread obtained the most similar level of quality to the control sample.

## Figures and Tables

**Table 1:** Recipes of mixes based on 1 kg of flour.

Ingredients	Amount [g]			
	Control	Flour with BH	Flour with BR	Flour with FL
Rye flour type 720	667	600	600	600
Wheat flour type 650	333	300	300	300
Buckwheat fiber (BH)	-	100	-	-
Beetroot fiber (BR)	-	-	100	-
Flax fiber (FL)	-	-	-	100

**Table 2:** Selected quality indicators of flour and dough samples.

Samples	Flour Moisture [%]	Protein Content [%]	Ash Content [%]	Falling Number [s]	Water Absorption [%]	Properties of Dough			
						Development Time [min]	Stability [min]	Degree of Softening [FU]	Farinograph Quality Number
Control	12.68 ± 0.2 ^b^	10.6 ^c^	0.89 ^d^	163 ± 4 ^a^	58.5 ± 0.0 ^c^	4.2 ± 0.5 ^ab^	3.5± 0.4 ^b^	94 ± 7 ^d^	64 ± 6 ^ab^
Dough with BH	12.31 ± 0.0 ^a^	9.3 ^d^	2.76 ^a^	234 ± 2 ^c^	55.6 ± 0.0 ^a^	3.2 ± 0.9 ^a^	4.0± 0.0 ^bc^	68 ± 1 ^c^	71 ± 3 ^b^
Dough with BR	12.38 ± 0.1 ^a^	11.8 ^b^	1.57 ^b^	227 ± 2 ^b^	56.5 ± 0.0 ^b^	5.0 ± 0.4 ^b^	4.4± 0.0 ^c^	60 ± 1 ^a^	76 ± 0 ^c^
Dough with FL	12.31 ± 0.2 ^a^	13.3 ^a^	1.36 ^c^	244 ± 4 ^d^	60.3 ± 0.1 ^d^	4.2 ± 0.2 ^a^	1.4 ± 0.4 ^a^	64 ± 11 ^b^	60 ± 6 ^a^

Parameters in columns denoted with the same letters do not differ significantly at the level of confidence α = 0.05. FU—farinograph unit; water absorption—percentage of water required to yield a dough consistency of 500 BU; development time—time to reach a maximum consistency; stability—time at which dough consistency is 500 BU; degree of softening—the difference between the maximum of the curve and the value after 12 min; farinograph quality number—determined by the farinograph software based on the values of the other farinographic parameters; its value shows the general quality of the flour, the maximum being 200, the minimum 0, but for flour of good baking quality it is often between 50 and 100.

**Table 3:** Pasting characteristics of flour samples.

Samples	T_0_ [°C]	η_max_ [BU]	T_η max_ [°C]	η_95ᵒC_ [BU]	BD [BU]	SB [BU]	η_30°C_ [BU]
Control	56.0 ± 0.0 ^b^	295.0 ± 1.4 ^b^	71.8 ± 0.6 ^a^	31.0 ± 1.4 ^a^	269.0 ± 2.8 ^b^	53.5 ± 0.7 ^a^	79.5 ± 2.1 ^a^
Flour with BH	58.1 ± 0.2 ^d^	234.0 ± 12.7 ^a^	74.5 ± 0.8 ^b^	47.0 ± 0.0 ^b^	192.5 ± 13.4 ^a^	123.5 ± 2.1 ^c^	165.0 ± 1.4 ^c^
Flour with BR	57.1 ± 0.1 ^bc^	234.0 ± 7.1 ^a^	72.3 ± 0.2 ^a^	36.0 ± 4.2 ^a^	204.0 ± 4.2 ^a^	65.0 ± 1.4 ^b^	95.0 ± 4.2 ^b^
Flour with FL	55.4 ± 0.0 ^a^	656.0 ± 3.5 ^c^	73.1 ± 0.6 ^ab^	116.5 ± 2.1 ^c^	550.5 ± 6.4 ^c^	397.5 ± 4.9 ^d^	503.5 ± 7.8 ^d^

Parameters in columns denoted with the same letters do not differ significantly at the level of confidence α = 0.05.

**Table 4:** Parameters of power-law model of rye–wheat dough samples.

Sample	$G^{\prime }\left(\omega \right)=K^{\prime }\cdot \omega^{n^{\prime }}$			$G^{\prime \prime }\left(\omega \right)=K^{\prime \prime }\cdot \omega^{n^{\prime \prime }}$			tg δ*
	K′ [Pas^n′^]	n′ [-]	R^2^	K″ [Pas^n″^]	n″ [-]	R^2^	
Control	22,399 ± 702 ^a^	0.21 ± 0.01 ^a^	0.997	8774 ± 396 ^a^	0.16 ± 0.01 ^a^	0.977	0.44 ± 0.01 ^c^
Dough with BH	23,395 ± 502 ^ab^	0.21 ± 0.00 ^a^	0.996	8672 ± 370 ^a^	0.17 ± 0.01 ^b^	0.988	0.41 ± 0.01 ^a^
Dough with BR	25,361 ± 1683 ^b^	0.21 ± 0.01 ^a^	0.996	9697 ± 615 ^b^	0.16 ± 0.01 ^a^	0.976	0.42 ± 0.00 ^b^
Dough with FL	37,471 ± 1378 ^c^	0.20 ± 0.01 ^a^	0.996	13,590 ± 307 ^c^	0.16 ± 0.00 ^a^	0.975	0.40 ± 0.01 ^a^

Parameters in columns denoted with the same letters do not differ significantly at the level of confidence α = 0.05.

**Table 5:** Parameters of Burger’s model of dough samples.

Sample	J_0_(×10^3^) [Pa^−1^]	J_1_(×10^3^) [Pa^−1^]	λ_ret_ [s]	η_0_(×10^−5^) [Pa s]
Control	0.107 ± 0.02 ^c^	0.248 ± 0.09 ^b^	129.4 ± 35.2 ^a^	15.2 ± 4.5 ^a^
Dough with BH	0.080 ± 0.01 ^b^	0.131 ± 0.02 ^a^	95.0 ± 16.7 ^a^	12.8 ± 0.2 ^a^
Dough with BR	0.082 ± 0.01 ^b^	0.148 ± 0.03 ^a^	106.7 ± 21.4 ^a^	13.4 ± 1.9 ^a^
Dough with FL	0.053 ± 0.01 ^a^	0.107 ± 0.02 ^a^	124.3 ± 22.3 ^a^	27.7 ± 5.7 ^b^

Parameters in columns denoted with the same letters do not differ significantly at the level of confidence α = 0.05.

**Table 6:** Parameters of the laboratory baking of the rye–wheat breads with fiber preparations.

Sample	Dough Yield [%]	Baking Loss [%]	Bread Yield [%]	Loaf Volume [cm^3^]	Specific Volume [cm^3^/g]
Control	164.97 ± 0.67 ^b^	9.51 ± 0.42 ^c^	139.43 ± 0.48 ^a^	430 ± 0 ^d^	2.04 ± 0.00 ^c^
Bread with BH	163.58 ± 0.16 ^a^	8.71 ± 0.18 ^b^	140.39 ± 0.11 ^a^	420 ± 7 ^c^	2.03 ± 0.02 ^c^
Bread with BR	164.78 ± 0.31 ^b^	8.49 ± 0.22 ^a^	142.18 ± 0.68 ^b^	405 ± 7 ^a^	1.87 ± 0.03 ^a^
Bread with FL	167.82 ± 0.05 ^c^	9.41 ± 0.48 ^c^	144.67 ± 0.90 ^c^	415 ± 7 ^b^	1.93 ± 0.04 ^b^

Parameters in columns denoted with the same letters do not differ significantly at the level of confidence α = 0.05.

**Table 7:** Color parameters of bread crumb and crust.

	Crumb			Crust		
Sample	L*	a*	b*	L*	a*	b*
Control	60.2 ± 0.8 ^d^	3.5 ± 0.1 ^a^	18.6 ± 0.4 ^d^	51.8 ± 09 ^d^	17.8 ± 0.5 ^c^	31.8 ± 0.6 ^d^
Bread with BH	35.3 ± 0.7 ^b^	3.8 ± 0.0 ^a^	8.3 ± 0.0 ^a^	38.1 ± 1.8 ^b^	5.6 ± 0.2 ^a^	11.2 ± 0.4 ^a^
Bread with BR	33.3 ± 0.7 ^a^	24.1 ± 0.7 ^c^	17.2 ± 0.3 ^c^	32.0 ± 0.9 ^a^	20.5 ± 0.7 ^d^	12.8 ± 0.4 ^b^
Bread with FL	52.9 ± 0.3 ^c^	4.7 ± 0.1 ^b^	16.3 ± 0.2 ^b^	47.7 ± 0.2 ^c^	12.0 ± 0.1 ^b^	25.0 ± 0.3 ^c^

Parameters in columns denoted with the same letters do not differ significantly at the level of confidence α = 0.05.

**Table 8:** Texture parameters of bread crumb.

Sample	Hardness [N]	Cohesiveness [-]	Chewiness [N]	Gumminess [N]	Resilience [-]
Control	22.7 ± 1.1 ^a^	0.4 ± 0.1 ^a^	4.0 ± 0.9 ^a^	9.4 ± 1.1 ^a^	0.16 ± 0.0 ^a^
Bread with BH	33.5 ± 4.1 ^b^	0.4 ± 0.0 ^a^	5.2 ± 0.8 ^ab^	12.4 ± 1.5 ^b^	0.15 ± 0.0 ^a^
Bread with BR	35.1 ± 3.7 ^b^	0.4 ± 0.0 ^a^	6.0 ± 1.7 ^b^	14.4 ± 2.1 ^b^	0.16 ± 0.0 ^a^
Bread with FL	32.6 ± 2.4 ^b^	0.4 ± 0.1 ^a^	4.4 ± 0.9 ^ab^	11.7 ± 2.0 ^ab^	0.15 ± 0.0 ^a^

Parameters in columns denoted with the same letters do not differ significantly at the level of confidence α = 0.05.

**Table 9:** Sensory evaluation of breads.

Sample	Appearance	Crumb	Crust	Flavor	Total Score	Quality Level
Control	5.0 ± 1.0 ^a^	7.0 ± 0.9 ^a^	9.0 ± 1.0 ^a^	6.0 ± 1.1 ^a^	27.0 ± 1.1 ^a^	II
Bread with BH	4.0 ± 1.1 ^b^	6.0 ± 0.5 ^b^	6.0 ± 1.0 ^c^	3.0 ± 1.0 ^c^	19.0 ± 1.5 ^c^	III
Bread with BR	4.0 ± 0.9 ^b^	6.0 ± 1.1 ^b^	9.0 ± 0.8 ^a^	5.0 ± 1.2 ^b^	24.0 ± 1.8 ^b^	II
Bread with FL	4.0 ± 0.4 ^b^	2.0 ± 1.5 ^c^	7.0 ± 0.6 ^b^	5.0 ± 1.0 ^b^	18.0 ± 1.0 ^c^	III

Parameters in columns denoted with the same letters do not differ significantly at the level of confidence α = 0.05.

## Data Availability

Data is contained within the article.
